# Metabolomics Analysis Reveals the Accumulation Patterns of Flavonoids and Volatile Compounds in *Camellia oleifera* Petals with Different Color

**DOI:** 10.3390/molecules28217248

**Published:** 2023-10-24

**Authors:** Haitao Zeng, Mengjiao Chen, Tao Zheng, Qi Tang, Hao Xu

**Affiliations:** Shaanxi Province Key Laboratory of Bio-Resources, Qinba Mountain Area Collaborative Innovation Center of Bioresources Comprehensive Development, Qinba State Key Laboratory of Biological Resources and Ecological Environment (Incubation), School of Biological Science and Engineering, Shaanxi University of Technology, Hanzhong 723001, China; zenghaitao@snut.edu.cn (H.Z.); cmj18168212780@163.com (M.C.); tangqi@snut.edu.cn (Q.T.); xh2003@126.com (H.X.)

**Keywords:** *Camellia oleifera* petal, flavonoids, volatile organic compounds, UPLC–MS/MS, HS–SPME–GC–MS

## Abstract

To systematically and comprehensively investigate the metabolic characteristics of coloring substances and floral aroma substances in *Camellia oleifera* petals with different colors, ultrahigh-performance liquid chromatography–mass spectrometry (UPLC–MS/MS) and headspace solid phase microextraction and gas chromatography–mass spectrometry (HS–SPME–GC–MS) metabolomics methods were applied to determine the metabolic profiles of white, candy-pink and dark-red petals. The results revealed that 270 volatile organic compounds were detected, mainly terpenoids, heterocyclic, esters, hydrocarbons, aldehydes, and alcohols, in which phenylethyl alcohol, lilac alcohol, and butanoic acid, 1-methylhexyl ester, hotrienol, alpha-terpineol and 7-Octen-4-ol, 2-methyl-6-methylene-, (S)-, butanoic acid, 2-methyl-, 2-methylbutyl ester, 2,4-Octadienal, (E,E)- could act as the floral scent compounds. A total of 372 flavonoid compounds were identified, and luteolin, kaempferol, cyanidin and peonidin derivatives were considered as the main coloring substances for candy-pink and dark-red petal coloration. In conclusion, this study intuitively and quantitatively exhibited the variations in flower color and floral scent of *C. oleifera* petal with different colors caused by changes in variations of flavonoids and volatile organic compound composition, and provided useful data for improving the sensory quality and breeding of *C. oleifera* petals.

## 1. Introduction

*Camellia oleifera*, belonging to Camellia in Theaceae, is a unique woody oil plant in China with high oil content [1]. *C. oleifera* flowers are complete flowers with high ornamental value [2]. The petals are generally white, and a few are pink or red, with unique delicate fragrance and sweetness [3]. *C. oleifera* petals are rich in phenolic compounds and volatile compounds, including phenolic acids, flavonoids, alcohols, ketones, aromatic hydrocarbons, esters and aldehydes, which are the most important secondary metabolites [4,5]. In addition, *C. oleifera* petals are abundant in biochemical components such as amino acids, proteins, tea polysaccharides and saponins, which have significant antioxidant, lipid-lowering, anti-allergy, hypoglycemic, gastrointestinal protection and immunity-enhancement functions [6].

Petal color is the typical target of research in ornamental plants, as flowers attract pollinators, absorb ultraviolet (UV) radiation, and decorate the environment [7,8]. Petals’ color diversity is mainly caused by the pigment types and contents [9]. Pigment compounds are widely distributed in plant tissues, and the three main types controlling petal color are flavonoids, carotenoids and betaines [10,11]. *Camellia oleifera* petals have been the subject of recent studies due to the plant’s status as an ornamental plant [12]. The pigments in *Camellia* petals are mainly flavonoids (especially anthocyanins), which are contributed to the formation of pink and red petals [7,13]. Cyanidin-core structure pigments (such as cyanidin 3,5-di-*O*-glucoside) have the potential to produce the most dominant phenotype among wild red-flowered *Camellia* species in China [14]. Cyanidin-3-O-(6″-*O*-malonyl) glucoside was the main anthocyanin component in the *Camellia japonica* petals, while cyanidin-3-*O*-rutinoside, peonidin-3-*O*-glucoside, cyanidin-3-Oglucoside, and pelargonidin-3-*O*-glucoside were responsible for the color intensity of the *C. japonica* petals [7]. Further research on these pigments will have important guiding significance for exploring the molecular regulation mechanism of flower color change in *C. oleifera*.

Flower fragrance is an important trait of ornamental plants and the main basis for evaluating the quality of flowers [15,16]. Volatile organic compounds (VOCs) emitted from flowers are mainly divided into three groups: terpenoids, phenylpropanoids/benzenoids, and fatty acid derivatives [17,18]. The content of volatile substances in *C. oleifera* petals is obviously different due to different flowering periods and varieties. Amounts of 22 fatty acid derivatives, 12 aromatic compounds and 2 terpenoids were identified from *C. oleifera* flowers, while the VOCs in nectar were mainly fatty acid derivatives. Amounts of 26, 36 and 48 aroma compounds were identified from *C. japonica*, *C. oleifera*, and *Camellia sinensis*, in which eugenol and decylacetate were the characteristic compounds in *C. japonica* petal, dihydro-3-methyl-2(3H)-furanone and myrcene were the characteristic compounds in *C. oleifera* petal, and 6,10,14-trimethyl-2-pentadecanone and citral were the characteristic compounds in *C. sinensis* petal [19]. A total of 5 terpenes, 1 aromatic hydrocarbons, 1 alkanes and 1 esters were found in *Siraitia grosvenorii*, in which the terpenes occupied a relative content of 71.07%, ranking the highest content [20]. Robin et al. found that the contents of volatile substances such as linalool, geranyl, linalool oxide, acetophenone, pentadienal, benzaldehyde and caproic acid were higher in tea flower petals during the flowering phase [21]. The five most abundant compounds, including beta-cis-ocimene, cis linalool oxide, lilac alcohol, phenylethyl alcohol, and 6-ethenyldihydro-2,2,6-trimethyl-2H-Pyran-3(4 H)-one were identified from 22 species within genus *Camellia*. L-linaloo was the compound with the highest relative amount (75.94%) in the flowers of 12 *Camellia* species, followed by (Z)-3-Hexenyl acetate (42.48%), Heptan-2-one (31.67%), (Z)-3-Hexen-1-ol (23.79%), and (S)-2-Heptanol (20.95%) [22].

Petal color and fragrances in *C. oleifera* are very diverse, and flower color and scent have always been the important characters in the selection process [23,24]. However, the research on *C. oleifera* is mainly concentrated in seedling-raising techniques, cultivation and management techniques, stress resistance study and so on. The investigations on characteristics of key coloring substances and VOCs in *C. oleifera* petals have not been reported. Here, ultrahigh-performance liquid chromatography–mass spectrometry (UPLC–MS/MS) and headspace solid phase microextraction and gas chromatography–mass spectrometry (HS–SPME–GC–MS) were employed to identify and quantify the differential flavonoids and VOCs in *C. oleifera* petals with different color. The complete chemical characterization of flavonoids and VOCs in *C. oleifera* petals was established to understand the petals’ color and fragrance variations. The results laid a metabolic foundation for further revealing the flower color and the VOCs variations in *C. oleifera* petals, and provided valuable information for understanding the flower color and scent change mechanism and metabolic pathways in *C. oleifera* petals.

## 2. Results

### 2.1. Analysis of Metabolite Profiling in C. oleifera Petals with Different Color

**Figure 1:**
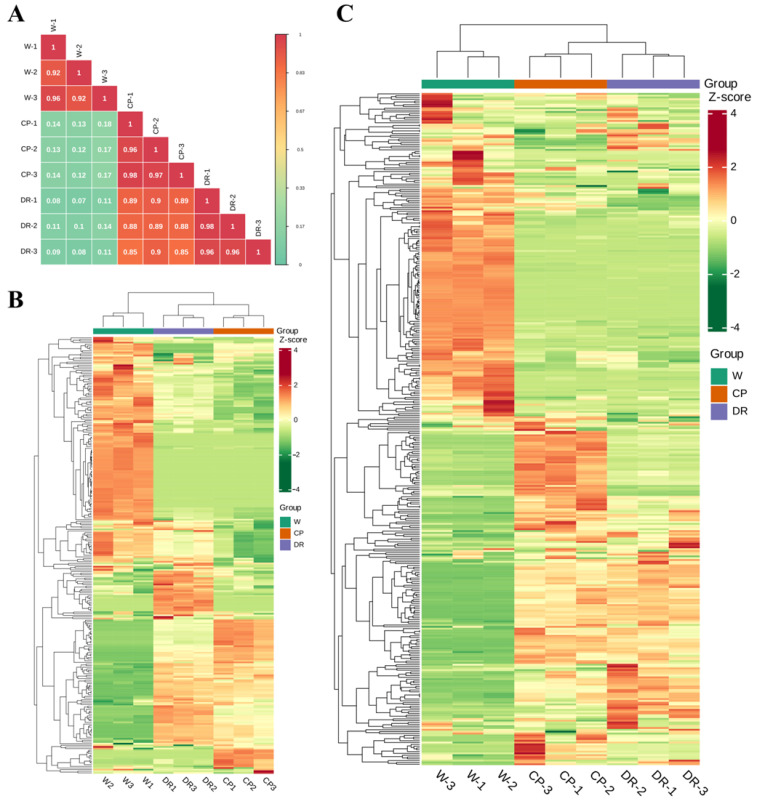
Differential petals chemotype in *C. oleifera* petals with different color, W = white petals, CP = candy-pink petal, DR = dark-red petal. (**A**): Correlation analysis of three *C. oleifera* petals based on identified VOCs and flavonoids; (**B**): Heatmap of the 270 VOCs identified in three *C. oleifera* petals with three biological replicates; (**C**): Heatmap of flavonoids identified in three *C. oleifera* petals with three biological replicates. The relative content values of all metabolites were denoted with a unique color, among which red color indicated a high accumulation level, and green color indicated a low accumulation level.

To further understand differences of flavonoids and volatile profiling in *C. oleifera* petals with different colors, the flavonoids and VOCs were determined by UPLC–MS/MS and HS–SPME–GC–MS. Pearson’s correlation coefficient r was applied to evaluate the biological repeatability of each group of the petal samples. When r^2^ was close to 1, the correlation between the two repeated samples was stronger, which indicated that the metabolite data had good homogeneity (Figure 1A).

Based on the quality evaluation, there were significant differences in the levels of VOCs among the W, CP, and DR petals (Figure 1B). A total of 270 VOCs were tentatively detected and identified, including 49 terpenoids, 47 heterocyclic compounds, 43 esters, 30 hydrocarbons, 27 aldehydes, 23 alcohols, 17 ketones, 8 amines, 6 phenols, 6 nitrogen compounds, 5 acids, 4 aromatics, 3 sulfur compounds, and 2 others (Table S1), which indicated that terpenoids, esters, heterocyclic compound, hydrocarbons, and alcohol were the main VOCs in *C. oleifera* petals. Moreover, the relative content of VOCs in W petals was significantly different from that in CP and DR petals. Cluster analysis of the detected petal samples and metabolites indicated that there were significant differences in VOC accumulation patterns in the three petals samples, indicating that there were significant differences in the composition of the three petals (Figure 1B).

Qualitative and quantitative analysis of flavonoid metabolites in W, CP, and DR petals by UPLC-MS/MS was carried out, and a total of 372 flavonoid metabolite species were detected and quantified, including 26 proanthocyanidins, 8 biflavones, 38 tannins, 22 flavanols, 83 flavonols, 85 flavonoids, 27 anthocyanins, 11 dihydroflavonols, 27 flavanones, 6 aurones, 23 chalcones (including C-glucosylquinochalcones), and 9 others (Table S2). Nine samples could be clearly divided into three groups based on the HCA heatmap (Figure 1C). Interestingly, a clear separation was found between W, CP, and DR petals, demonstrating that the accumulation of flavonoids in the three petals were obviously distinct. The above statistical results demonstrated that all petal samples had good repeatability, and the data of flavonoid metabolites were reliable, and were suitable for further qualitative and quantitative analysis.

### 2.2. PCA and OPLS-DA of the Three C. oleifera Petals with Different Colors

**Figure 2:**
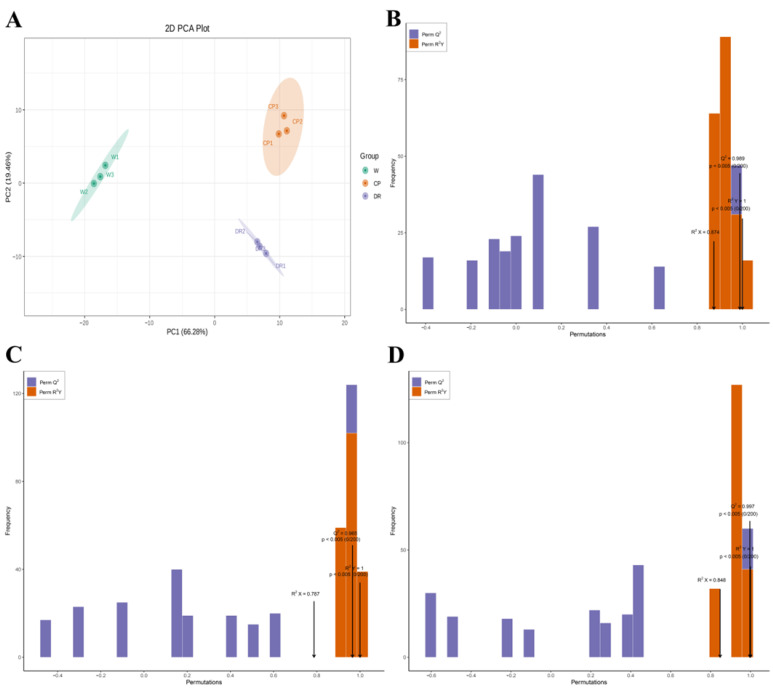
The 2D-PCA plot and OPLS-DA plot of the three *C. oleifera* petals based on the relative content of flavonoids and VOCs. (**A**): The 2D-PCA score plot of W, CP, and DR petals; (**B**): Score plots of the OPLS-DA model for W_vs_CP; (**C**): Score plots of the OPLS-DA model for CP_vs_DR; (**D**): Score plots of the OPLS-DA model for W_vs_DR. W = white petals; CP = candy-pink petal; DR = dark-red petal.

In the 2D-PCA score plot, the results exhibited that the flavonoid and VOCs metabolites in three petals displayed a clear separation trend between groups, and there was a tight aggregation trend within groups. It could be seen that the cumulative contribution rate of two principal components (PC1 66.28% × PC2 19.46%) reached 85.74% (Figure 2A). W, CP, and DR petals were obviously separated, and the biologically repeated same petals were closely grouped. These results indicated that the volatile profiling data had high repeatability, which was convenient for further analysis.

The OPLS-DA model was applied to compare the differential accumulation metabolites (DAMs) in the three *C. oleifera* petals with different colors. When high predictability (Q^2^) > 0.9 and the goodness of fit was strong (R^2^X, R^2^Y was close to 1), the model was considered to be excellent and stable. Here, the high Q^2^, R^2^X, and R^2^Y were obtained to evaluate the validity of OPLS-DA model between the three *C. oleifera* petals. All the flavonoids and VOCs in three *C. oleifera* petals were accessed to determine the difference in the W_vs_CP comparison (Q^2^ = 0.989, R^2^X = 0.874, and R^2^Y = 1.000; Figure 2B), CP_vs_DR comparison (Q^2^ = 0.965, R^2^X = 0.787, and R^2^Y = 1.000; Figure 2C), and W_vs_DR comparison (Q^2^ = 0.997, R^2^X = 0.848, and R^2^Y = 1.000; Figure 2D) based on the OPLS-DA model through pairwise comparison. The results of OPLS–DA analysis and cross validation demonstrated that the Q^2^ of the three comparison groups was greater than 0.9, indicating that the model was accurate and stable, which explained the variations of flavonoids and VOCs in the three *C. oleifera* petals and could be performed to further screen the DAMs among different comparison groups using VIP values.

### 2.3. Differential Accumulation Metabolites of Volatile Organic Compounds Analysis of C. oleifera Petals

#### 2.3.1. Differentially Accumulated Metabolites of Volatile Organic Compounds

**Figure 3:**
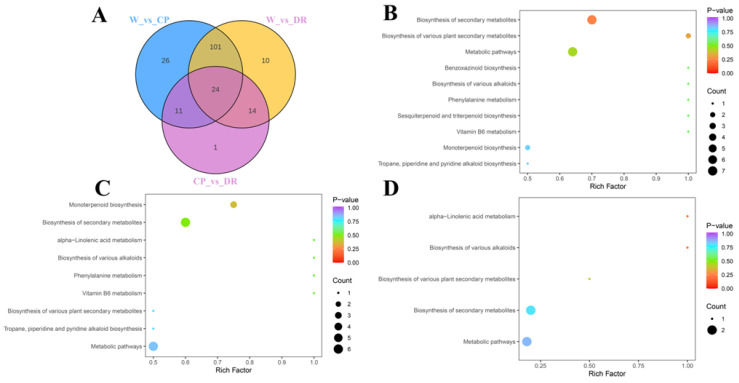
Venn diagram and pathway analysis of VOCs-DAMs in three *C. oleifera* petals. (**A**): The Venn diagram results among W_vs_CP, W_vs_DR, and CP_vs_DR comparisons. (**B**–**D**): KEGG pathway enrichment of the VOCs-DAMs of the W_vs_CP, W_vs_DR, and CP_vs_DR comparison, respectively. W = white petals; CP = candy-pink petal; DR = dark-red petal.

To screen the differential accumulation metabolites of volatile organic compounds (VOCs-DAMs) among three *C. oleifera* petals, the 270 VOCs metabolites were selected based on the one-dimensional analysis *t* test (*p* < 0.05), multidimensional analysis of VIP value (VIP > 1) of OPLS-DA, and fold change of ≥2 or ≤0.5, and there were 180 VOCs-DAMs in the W_vs_CP_vs DR comparison (Table S3). There were 162 VOCs-DAMs (74 up-regulated, and 88 down-regulated) in the W_vs_CP comparison (Table S4), 149 VOCs-DAMs (79 up-regulated, and 70 down-regulated) in the W_vs_DR comparison (Table S5), 50 VOCs-DAMs (21 up-regulated, and 29 down-regulated) in the CP_vs_DR comparison (Table S6). Next, VOCs-DAMs in the three comparison groups (W_vs_CP, W_vs_DR, and CP_vs_DR) were classified into 11, 12, and 10 different categories, respectively, and the most common VOCs-DAMs were esters, hydrocarbons, terpenoids, and alcohols. In addition, 24 VOCs-DAMs were identified among the three groups, indicating that these 24 VOCs-DAMs were differentially accumulated among the three *C. oleifera* petals (Figure 3A).

Enrichment analysis of VOCs-DAMs in three *C. oleifera* petals samples was performed by KEGG to obtain comprehensive functional information, and Most of the VOCs-DAMs were categorized into metabolic pathways, biosynthesis of secondary metabolites, alpha-Linolenic acid metabolism, biosynthesis of various alkaloids, and biosynthesis of various plant secondary metabolites (Figure 3B–D).

#### 2.3.2. Crucial Differential VOCs Related to *C. oleifera* Petals Aroma

**Figure 4:**
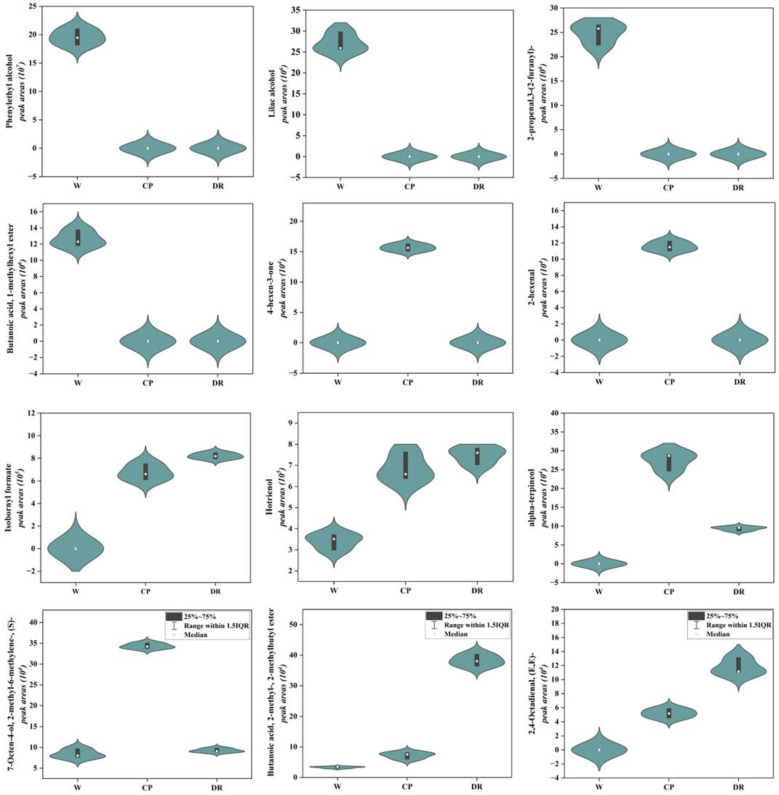
Violin plots of peak areas values of 12 crucial differential VOCs identified in W, CP, and DR petals. W = white petals; CP = candy-pink petal; DR = dark-red petal. The distribution and probability density of the 12 VOCs-DAMs were represented by a combination of box plots and density plots. The outer shapes represented the density of the peak area values distribution, the black rectangular box in the middle represents the quartile range, and the white circle in the middle represents the median.

As shown in Figure 4, the phenylethyl alcohol, lilac alcohol, 2-propenal,3-(2-furanyl)-, and butanoic acid, 1-methylhexyl ester were only detected in W petals, and displayed a higher accumulation level. 4-hexen-3-one and 2-hexenal were found only in CP petals with a higher level. Isobornyl formate, and hotrienol were present at a higher level in DR petals. In the W_vs_CP comparison, most VOCs-DAMs were terpenoidsthe. The top five up-regulated VOCs with higher content in CP petals were Butanoic acid, 3-hexenyl ester, (E)- (S)- (4.15-fold), 7-Octen-4-ol, 2-methyl-6-methylene-, (S)- (4.08-fold), (E)-2,6-Dimethylocta-5,7-dien-2-ol (3.54-fold), cyclohexene, 1-methyl-4-(1-methylethylidene)- (2.73-fold), and hotrienol (2.02-fold). Lilac aldehyde (0.132-fold), tetradecane (0.131-fold), isopinocarveol (0.106-fold), gamma-terpinene (0.059-fold), and undecane, 3,5-dimethyl- (0.007-fold) were significantly down-regulated from W petal to CP petal. In the W_vs_DR comparison, the higher concentrations in the DR petals were furan, 3-(4-methyl-3-pentenyl)-, hotrienol, 2-isopropyl-5-methylhex-2-enal, 1-Nonen-4-ol, Cyclohexene, 1-methyl-4-(1-methylethylidene)-, 3-hexen-1-ol, propanoate, (Z)-, and butanoic acid, 2-methyl-, 2-methylbutyl ester, which were up-regulated by 2.05-, 2.20-, 2.58-, 2.38-, 2.35-, 2.68-, and 11.49-fold, respectively. Undecane, 3,5-dimethyl-, gamma-terpinene, and (3R,6R)-2,2,6-trimethyl-6-vinyltetrahydro-2H-pyran-3-ol were up-regulated by 0.009-, 0.058-, and 0.082-fold, respectively.

In the CP_vs_DR comparison, it was found that butanoic acid, 2-methyl-, 2-methylbutyl ester, 2,4-Octadienal, (E,E)-, and 1H-Imidazole, 2-propyl- displayed a high accumulation level in the DR petals, which were up-regulated by 5.28-, 2.26- and 2.99-fold, respectively. 7-Octen-4-ol, 2-methyl-6-methylene-, (S)- and alpha-terpineol were down-regulated by 0.269- and 0.341-fold, respectively.

Overall, the contents of phenylethyl alcohol, lilac alcohol, and butanoic acid, 1-methylhexyl ester with rose, fruity, and honey aroma attributes in white *C. oleifera* petals were significantly higher than those in candy-pink and dark-red petals, which might make the white petals richer in aroma. The hotrienol, alpha-terpineol and 7-Octen-4-ol, 2-methyl-6-methylene-, (S)- with sweet and delicate fragrance flavor exhibited a higher accumulation level in candy-pink *C. oleifera* petals. The hotrienol, butanoic acid, 2-methyl-, 2-methylbutyl ester, and 2,4-Octadienal, (E,E)- with wood and fruity flavor were the primary VOCs in the dark-red *C. oleifera* petals.

### 2.4. Differential Flavonoid Compounds Analysis of C. oleifera Petals

#### 2.4.1. Differentially Accumulated Metabolites of Flavonoid Compounds 

**Figure 5:**
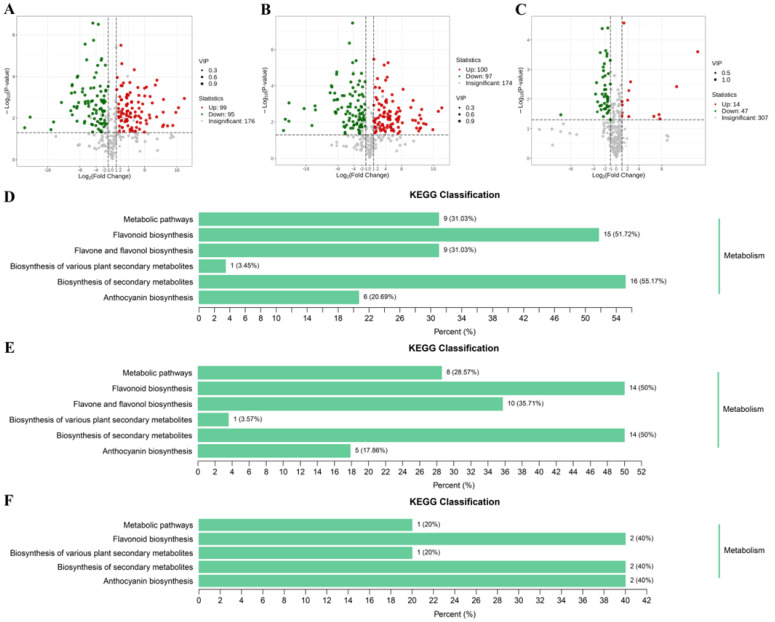
(**A**–**C**): Volcano plots of DAMs in W_vs_CP, W_vs_DR, and CP_vs_DR comparisons; red indicated up-regulated differential metabolites, and blue indicated down-regulated differential metabolites; (**D**–**F**): KEGG pathway enrichment analysis of the DAMs in W_vs_CP, W_vs_DR, and CP_vs_DR comparisons.

*C. oleifera* petals were found to be rich in flavonoids, and their flavonoid contents were markedly influence by color genotypes. In our study, the differentially accumulated metabolites (DAMs) between pairwise comparisons among W_vs_CP, W_vs_DR, and CP_vs_DR were screened by the variable importance in projection values (VIP) ≥ 1 and fold change ≥ 2 or fold change ≤ 0.5. The W_vs_CP comparison and W_vs_DR comparison had the largest number of up-regulated and down-regulated DAMs (Figure 5). Among these comparisons, there were 194 DAMs (99 up-regulated and 95 down-regulated) in the W_vs_CP comparison (Figure 5A), 197 DAMs (100 up-regulated and 97 down-regulated) in the W_vs_DR comparison (Figure 5B), and 61 DAMs (14 up-regulated and 47 down-regulated) in the CP_vs_DR comparison (Figure 5C), respectively. KEGG pathway enrichment analyses were carried out to gain further insights into the biochemical pathway to which the DAMs belonged to. The top three enriched KEGG pathways between the three comparisons were anthocyanin biosynthesis, flavonoid biosynthesis, and flavone and flavonol biosynthesis (Figure 5D–F). Given the role of anthocyanins and flavonoids in petal coloration, we deduced that the DAMs in anthocyanin biosynthesis pathway and flavonoid biosynthesis pathway might be likely the key metabolites underlying the variations in *C. oleifera* petals.

#### 2.4.2. Crucial Differential Compounds Related to *C. oleifera* Petals Color

**Figure 6:**
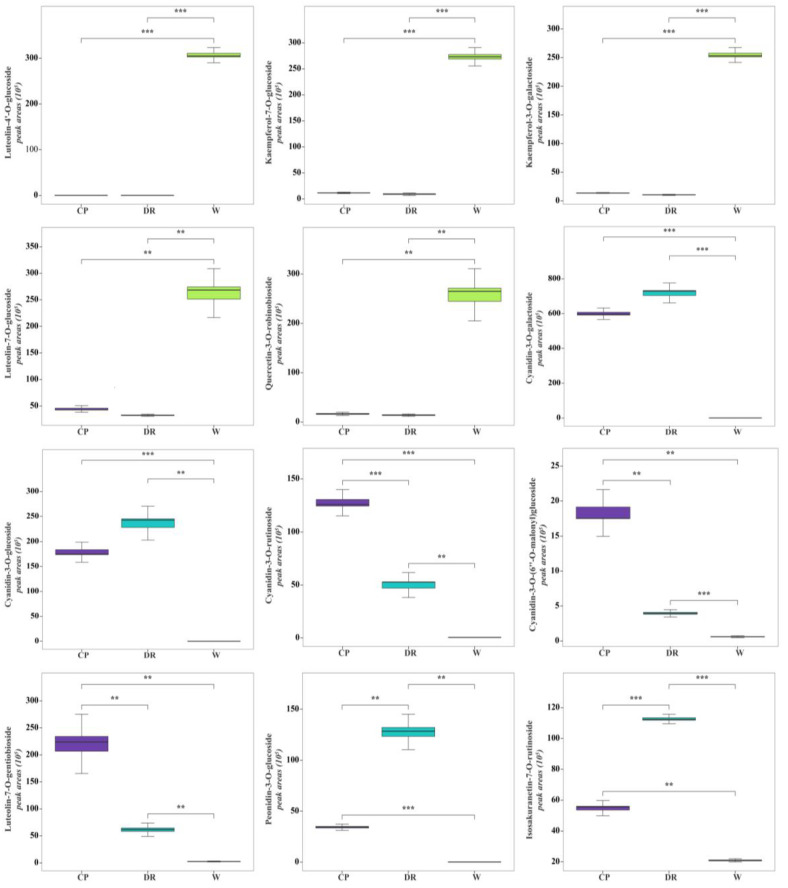
Histogram of peak areas of 12 differentially accumulated metabolites identified in W, CP, and DR petals. W = white petals; CP = candy-pink petal; DR = dark-red petal. Duncan’s test was applied to evaluate the significant difference among petals with different color. ** indicated the significant difference at the *p* < 0.05 level; *** indicated the significant difference at *p* < 0.01 level.

In the W petals, the concentrations of luteolin-4′-*O*-glucoside, kaempferol-7-*O*-glucoside, luteolin-7-*O*-glucoside, quercetin-3-*O*-robinobioside, and kaempferol-3-*O*-galactoside were significantly higher than those in CP and DR petals (Figure 6). In the W_vs_CP comparison, flavonols and anthocyanins accumulated to significantly higher levels in CP petals compared to W petals, especially cyanidin-3-*O*-galactoside, luteolin-7-*O*-gentiobioside, cyanidin-3-*O*-glucoside, cyanidin-3-*O*-rutinoside, kaempferol-3-*O*-glucorhamnoside, and kaempferol-3-*O*-glucoside-7-*O*-rhamnoside, which were up-regulated by 1596.79-, 70.58-, 6475.94-, 260.10-, 88.10, and 63.58-fold, respectively. In the W_vs_DR comparison, the top three most differentially abundant anthocyanins and flavanones with higher concentrations were cyanidin-3-*O*-galactoside, cyanidin-3-*O*-glucoside, peonidin-3-*O*-glucoside, and isosakuranetin-7-*O*-rutinoside, which were up-regulated by 1901.72-, 8447.98-, 1290.22, and 5.36-fold, respectively. In the CP_vs_DR comparisons, peonidin-3-*O*-glucoside and isosakuranetin-7-*O*-rutinoside in DR petals were 4.63- and 2.07-fold higher than that in CP petals, while cyanidin-3-*O*-(6″-*O*-malonyl)glucoside and luteolin-7-*O*-gentiobioside were decreased by 0.195- and 0.329-fold, respectively. However, the cyanidin-3-*O*-galactoside and cyanidin-3-*O*-glucoside with higher concentrations had no significant difference between CP and DR petals, which indicated that cyanidin-3-*O*-galactoside and cyanidin-3-*O*-glucoside were responsible for the coloration in CP and DR petals.

Accordingly, luteolin-4′-*O*-glucoside, kaempferol-7-*O*-glucoside, luteolin-7-*O*-glucoside, quercetin-3-*O*-robinobioside, and kaempferol-3-*O*-galactoside might be the key coloring substances for white color formation in *C. oleifera* petals. Cyanidin-3-*O*-glucoside, cyanidin-3-*O*-galactoside, cyanidin-3-*O*-rutinoside, cyanidin-3-*O*-(6″-*O*-malonyl)glucoside, luteolin-7-*O*-gentiobioside, peonidin-3-*O*-glucoside, and isosakuranetin-7-*O*-rutinoside were speculated to lead to the candy-pink and dark-red petal coloration, among which cyanidin-3-*O*-(6″-*O*-malonyl)glucoside and luteolin-7-*O*-gentiobioside were the key metabolites for CP petals coloration, and peonidin-3-*O*-glucoside and isosakuranetin-7-*O*-rutinoside were the main pigments contributing to dark-red coloration.

## 3. Discussion

*Camellia chekiangoleosa* with a bright flower color and beautiful flower type, and *Camellia yuhsienensis* with a heavy floral scent were the excellent garden greening and woody oil tree species. Flower color and floral fragrance, as important ornamental traits of plants, are the main indicators to identify and distinguish different varieties, and affect the ornamental value and economic value of plants [8,25,26]. However, the regulation mechanism on the formation of flower color and fragrance between different flower color varieties of *C. oleifera* is still unclear.

Flavonoids are one of the main pigment components involved in the formation of flower color [27]. The difference of composition and anthocyanin content directly affects the flower color of plants [28]. Previous studies have confirmed that cyanidin and its derivatives widely acted on red petals of plants [29]. For example, it was found that the content of cyanidin in *Rhododendron simsii Planch.* with red flower colors was the highest. Jin et al. (2018) had found that cyanidin was the main flavonoid component in rosa crimson glory, and its content was significantly higher than other substances [30]; it has also been found that the main pigment in the *Rosa rugosa* × *Rosa Sertata* was cyanidin-3-glucose [31]. Peonidin-3-*O*-glucoside and kaempferol derivatives were mainly detected from the red rapeseed petals [9]. The white petals of rosa glaucca pourr contained only flavonoids, while the pink petals and purple petals of rosa glaucca pourr contained flavonoids and anthocyanins [32]. White *Rosa multiflora* petal and white chrysanthemum petals only contain light yellow or near colorless pigments, such as flavonoids and flavonols [33]. Therefore, the difference of flavonols, flavones, and anthocyanin content will direct influence on plant petals color. Here, the qualitative and quantitative analysis of flavonoids and anthocyanins in white, candy-pink, and dark-red color petals of three *C. oleifera* cultivars was identified and detected by UPLC–MS/MS in this study. In the white petals, luteolin-4′-*O*-glucoside, kaempferol-7-*O*-glucoside, luteolin-7-*O*-glucoside, quercetin-3-*O*-robinobioside, and kaempferol-3-*O*-galactoside were the main pigments in white petals, which was in accordance with the results that luteolin and kaempferol were the main substances that determined the white color of ‘Rosa alba’ [34]. In the candy-pink and dark-red petals, cyanidin-3-*O*-glucoside, cyanidin-3-O-galactoside, cyanidin-3-*O*-rutinoside, cyanidin-3-*O*-(6″-*O*-malonyl)glucoside, and peonidin-3-*O*-glucoside displayed higher accumulation levels, which was consistent with this conclusion that those above pigments were the main anthocyanin components in red petals of *C.japonica* [35]. Moreover, the combination of flavonoids and other related co-pigment compounds with anthocyanins exhibited a hyperchromic effect, and the co-color effects also increased with the increase in anthocyanin methylation and glycosylation, of which the flavonol and flavonoids are the most common co-pigments [36]. Luteolin-7-*O*-gentiobioside and isosakuranetin-7-*O*-rutinoside were found at higher accumulation levels in candy-pink and dark-red petals, respectively, which might be combined with cyanidin and peonidin derivatives to form a co-pigmentation effect to make the petals pinker and redder. Therefore, luteolin, kaempferol, cyanidin, and peonidin derivatives were acted as the main pigments for *C. oleifera* petal coloration.

Floral scent is a mixture of chemical compounds emitted by plant tissues, which is a crucial factor that affects the overall aroma and consumer preference [37]. The petal flavor is strongly influenced by the type and amount of aroma components present [38]. VOCs are the main components of flowers, and the flowers of different plant varieties contain different VOCs [39]. For example, the main VOCs in ‘Gesang Lv’ were linalool, caryophyllene, oxidized linalool, and pinene, the main VOCs in ‘Gesang Hong’ included macrophyllene D, methyl decanoate, hexol, and pinene, and the main VOCs in ‘Gesang Huang’ were linalool, caryophyllene, methyl decanoate, and germacrene [40]. The volatile components of the flowers within the genus *Chimonanthus* were mainly terpenes and esters, and terpenoids and aromatic hydrocarbons were the main VOCs in rose [41]. A total of 270 volatile organic compounds were identified using HS–SPME in conjunction with GC–MS. Esters, hydrocarbons, terpenoids, and alcohols were the dominant aroma components in three *C. oleifera* petals in terms of quantity and proportion. Phenylethyl alcohol was the main aroma component, widely existing in plant essential oils, which is an important aroma compound, with rose, fruity, and honey flavor [42]. Here, phenylethyl alcohol was the main VOCs in the white petals, which was in accordance with the results that phenylethyl alcohol was the unique compound of ‘Fragrant cloud’ varieties and phenylethyl alcohol was the highest aroma components in *Rosa. odorata var odorata* [43]. The hotrienol and alpha-terpineol were detected with a high content in candy-pink petals. In accordance with our results, Liu et al. (2016) identified hotrienol and alpha-terpineol as the important aroma compounds in red freesia flower [44]. Zhang Ming et al. (2020) detected as main aroma constituent butanoic acid, 2-methyl-, 2-methylbutyl ester in *Areca catechu* flower [45], in which we detected a high accumulation of butanoic acid, 2-methyl-, 2-methylbutyl ester in the dark-red *C. oleifera* petal. Different volatile organic compounds and release amount were together contributed to the unique aroma of *C. oleifera* petals, which not only increased the ornamental value, but also had important significance for the commercial development of aromatic *C. oleifera* petals. In summary, phenylethyl alcohol, hotrienol, alpha-terpineol, and butanoic acid, 2-methyl-, 2-methylbutyl ester were generally present in white, candy-pink, and dark-red *C. oleifera* petal, which mainly contributed to the perfumery value.

Our understanding of the floral scent traits of *C. oleifera* petals with different colors is limited. A comprehensive knowledge of the coloring substances and volatile compounds in *C. oleifera* petals is the first step in cultivating colorful and aroma varieties. Esters, alcohols and terpenoids are ubiquitous in floral volatiles. Functional studies of genes involved in the biosynthesis of terpenoids would be very prospective. Finally, with the support of biotechnology tools, metabolic engineering methods for flower color and fragrance-related products could be realized. Therefore, our research not only would be helpful to provide comprehensive information about the characteristic components of flower color and fragrance in *C. oleifera* petals with different colors, but also lay a fundamental reference for cultivating excellent *C. oleifera* varieties with specific flower color and fragrance.

## 4. Materials and Methods

### 4.1. Materials

**Figure 7:**
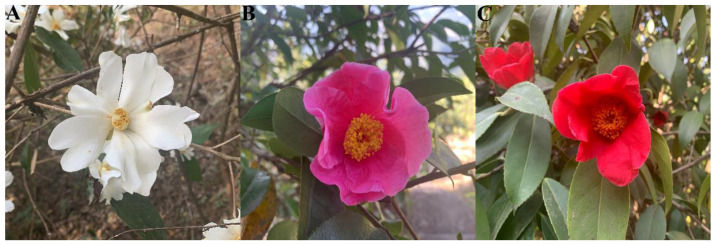
Petals colors of three *C. oleifera* varieties: (**A**): “*Camellia yuhsienensis*” (White, W); (**B**): “*Camellia semiserrata*” (Candy-pink, CP); (**C**): “*Camellia chekiangoleosa*” (Dark-red, DR).

Three *C. oleifera* varieties with different-colored petals were planted in the Shaanxi *C. oleifera* germplasm repository with similar soil conditions in Nanzheng District, Shaanxi Province, China. The petal colors of three *C. oleifera* varieties, namely “*Camellia yuhsienensis*” (White, W), “*Camellia semiserrata*” (Candy-pink, CP), and “*Camellia chekiangoleosa*” (Dark-red, DR) were white, candy-pink and dark-red, respectively (Figure 7). Three *C. oleifera* petals samples were collected from fresh petals during full flowering, with three biological replicates on 8 March 2023. Then, the petals were immediately stripped, put into liquid nitrogen, and brought back to the laboratory for storage at −80 °C for the flavonoid and volatile organic compound determination.

### 4.2. UPLC-MS/MS Analysis

#### 4.2.1. Petal Preparation and Extraction

Fresh petal samples were freeze-dried, and pulverized to powder with a grinder (MM 400, Retsch, Haan, Germany) at 30 Hz for 1.5 min. For each sample, 100 mg of powder was extracted in 1.0 mL of methanol. The extracts were vortexed once every 30 min for 30 s 6 times, and then put in the refrigerator at 4 °C overnight. After the sample was centrifuged (rotational speed 12,000 r·min^−1^, 10 min) at 4 °C for 3 min, the supernatant was absorbed, filtered by microporous filter membrane (pore size 0.22μm, Anpel Laboratory Technologies, Shanghai, China), and stored in the sample vial for UPLC-MS/MS analysis.

#### 4.2.2. UPLC–MS/MS Conditions

The abundances of flavonoid compounds were quantified with ultrahigh-performance liquid chromatography (UPLC)–mass spectrometry (MS/MS). The UPLC conditions were as follows: (1) SB-C18 column (1.8 µm, 2.1 mm × 100 mm, Agilent, Palo Alto, CA, USA); (2) column temperature: 40 °C; (3) flow rate: 0.35 mL per minute; (4) injection volume: 2.0 µL; (5) fluid phase: phase A is 0.1% formic acid solution, phase B is 0.1% formic acid acetonitrile solution; (6) elution procedure: 0 min, 95% A, 5% B; 0–9 min, 5% A, 95% B; 9–10 min, 5% A, 95% B; 10–11.1 min, 95% A, 5.0% B; 11.1–14 min, 95% A, 5.0% B. The quadrupole orbital trap mass spectrum conditions were as follows: (1) detection mode: positive ion mode (P+), 5500 V and negative ion mode, −4500 V; (2) ESI temperature: 550 °C; (3) GSI, 50 psi; GSII, 60 psi; the curtain gas, 25.0 psi; (4) collision-induced ionization parameter: high; (5) quality scanning range *m*/*z* 100–1200, scanning time 0.2 s; (6) a specific set of MRM transitions were monitored for each period according to the metabolites eluted within this period.

The metabolites were analyzed qualitatively by comparing the UPLC-MS/MS data with the local metabolite databases built by Met Ware, and the quantitative analysis of each metabolite was completed by MRM analysis of triple quadrupole mass spectrometry. And then, the mass spectrogram of all metabolites in petals with different color were obtained and the peak area of the mass spectrogram of each metabolite was integrated. Analyst 1.6.3 software (AB SCIEX, Concord, ON, Canada) was applied to process the UPLC-MS/MS data. The MultiaQuant™ software 3.0.3 (AB SCIEX, Concord, ON, Canada) was used to integrate and correct the chromatographic peaks, and the peak area of each chromatographic peak represented the relative content of the corresponding metabolite.

### 4.3. HS–SPME–GC–MS Analysis

#### 4.3.1. Petal Sample Preparation and Treatment

The fresh petal samples were ground to a powder in liquid nitrogen. An amount of 500 mg of the petal powder was immediately added to a 20 mL head-space vial (Agilent, Palo Alto, CA, USA) with NaCl saturated solution, and then the vials were sealed immediately and balanced for 5 min. Each vial was placed in 60 °C for 5 min for SPME analysis, then 120 μm Divinylbenzene (DVB)/Carboxen (CAR)/Polydimethylsiloxane (PDMS) extraction fibers (Agilent, CA, USA) were exposed to the headspace of the sample for 15 min at 60 °C. Desorption was performed at 250 °C at the injection port of GC-MS for 5 min.

#### 4.3.2. GC–MS Conditions

The GC–MS conditions were as follows: (1) column: DB-5MS capillary column (30 m × 0.25 mm, 0.25 μm, Agilent, Folsom, CA, USA); (2) shunt ratio: 1:15; (3) carrier gas: He (99.9999%); (4) flow rate: 1.2 mL/min; (5) injector temperature: 250 °C and detector: 280 °C; (6) temperature rise condition: 40 °C for 3.5 min, increasing at 10 °C/min to 100 °C, at 7 °C/min to 180 °C, at 25 °C/min to 280 °C, hold for 5 min. The MS conditions were as follows: (1): electron impact (EI) ionisation mode: 70 eV; (2): ion source temperature: 230 °C; (3) quadrupole mass detector: 150 °C; (4): transfer line temperature: 280 °C; (5): collection mode: Scan; (6) quality scanning range: *m*/*z* 40~400.

The mass spectrograms corresponding to each chromatographic peak were compared with NIST05 and NIST05s standard spectrum library (NIST Mass Spectral Database 2.2) to identify the volatile organic compounds in three *C. oleifera* petals. Each sample was repeated three times. The MassHunter quantitative software (version B.08.00, Agilent Technologies Inc., Santa Clara, CA, USA) was carried out to integrate and correct the chromatographic peaks, and peak area normalization method was applied to determine the relative content of each component after detection by GC-MS. The peak area of each chromatographic peak represented the relative content of the corresponding metabolite.

### 4.4. Qualitative and Quantitative Analyses of Metabolites

Orthogonal partial least squares discriminant (OPLS–DA) analysis was conducted on the total metabolites between samples of different groups and metabolites within samples of different groups to investigate the differences of flavonoid metabolites among the petals of three *C. oleifera* petals. The screening standards for differential metabolites were (1) Variable importance in projection (VIP), VIP > 1; (2) significance threshold, *p* < 0.05; (3) fold change ≥ 2 or fold change ≤ 0.5. Through searching the KEGG (Kyoto Encyclopedia of Genes and Genomes) database, functional annotation analysis and metabolic pathway enrichment analysis were performed on metabolites with significantly different contents obtained from metabolomics analysis.

### 4.5. Statistical Analysis

Chemometric analyses such as hierarchical cluster analysis (HCA), correlation analysis (CA) and orthogonal partial least squares discriminant analysis (OPLS–DA) were performed to systematically analyze the difference of the flavonoid compounds and volatile organic compounds in three *C. oleifera* petals, which were generated by using R (http://www.r-project.org/ (accessed on 20 September 2023)). Variance of flavonoid compounds and volatile organic compounds among three petals was calculated and generated by using SPSS 26.0 for Windows (SPSS Inc., Chicago, IL, USA). The violin plots and histograms were drawn and generated by using the Origin Pro 2023 for statistics and computing (Origin Lab, Northampton, MA, USA).

## 5. Conclusions

In our study, the metabolic profiles of coloring substances and volatile organic compounds in three *C. oleifera* petals with different colors were systematically evaluated to explore the differences in metabolites based on the UPLC–MS/MS and HS–SPME–GC–MS approach. The results demonstrated that a total of 372 flavonoid compounds and 270 volatile organic compounds were detected in the *C. oleifera* petals, which visually revealed how changes in the composition of flavonoid compounds and volatile organic compounds affected the overall flower color and floral scent of *C. oleifera* petals. Among them, phenylethyl alcohol, lilac alcohol, and butanoic acid, and 1-methylhexyl ester were the major flavor substances in white *C. oleifera* petals; hotrienol, alpha-terpineol and 7-Octen-4-ol, 2-methyl-6-methylene-, (S)- were the main flavor substances in candy-pink *C. oleifera* petals; and hotrienol, butanoic acid, 2-methyl-, 2-methylbutyl ester, and 2,4-Octadienal, (E,E)- were the primary flavor substances in the dark-red *C. oleifera* petals. Luteolin and kaempferol derivatives might be the key coloring substances contributed to white color formation, and cyanidin and peonidin derivatives were considered as the main coloring substances for candy-pink and dark-red petal coloration. In summary, this study not only investigated the key metabolites that controlled the flower color and fragrance of *C. oleifera* petals, but also aided in evaluating the metabolic quality of *C. oleifera* petals by laying a solid foundation for further cultivating *C. oleifera* varieties with a specific color and strong fragrance.

## Data Availability

The datasets generated or analyzed in the current study are available from the corresponding author on reasonable request.

## Supplementary Materials

The following supporting information can be downloaded at: https://www.mdpi.com/article/10.3390/molecules28217248/s1, Table S1: A total of 270 volatile organic compounds tentatively detected and identified from three C. oleifera petal; Table S2: A total of 372 flavonoid metabolite tentatively detected and identified from three C. oleifera petal; Table S3: Differential accumulation metabolites of volatile organic compounds in the W_vs_CP_vs DR comparison; Table S4: Differential accumulation metabolites of volatile organic compounds in the W_vs_CP comparison; Table S5: Differential accumulation metabolites of volatile organic compounds in the W_vs_DR comparison; Table S6: Differential accumulation metabolites of volatile organic compounds in the CP_vs_DR comparison.
